# Comparison for Efficacy and Tolerability among Ten Drugs for Treatment of Parkinson’s Disease: A Network Meta-Analysis

**DOI:** 10.1038/srep45865

**Published:** 2017-04-04

**Authors:** Chuanjun Zhuo, Xiaodong Zhu, Ronghuan Jiang, Feng Ji, Zhonghua Su, Rong Xue, Yuying Zhou

**Affiliations:** 1 Department of Psychological Medicine, Wenzhou Seventh People’s Hospital, Wenzhou, 325005, Zhejiang, China; 2 Institute of Mental Health, Jining Medical University, Jining, 272067, Shandong, China; 3 Department of Psychological Medicine, Tianjin Mental Health Center, Tianjin Anding Hospital, Tianjin, 300222, China; 4 Department of Psychological Medicine, Tianjin Anning Hospital, Tianjin, 300222, China; 5 Department of Neurology, Tianjin Medical University General Hospital, Tianjin, 300075, China; 6 Department of Psychological Medicine, Chinese PLA (People’s Liberation Army) General Hospital, Beijing, 100853, China; 7 Department of Psychological Medicine, The Second Affiliated Hospital of Jining Medical College, Jining, 272100, Shandong, China; 8 Department of Neurology, Tianjin Huanhu Hospital, Tianjin Brain Center, Tianjin, 300350, China

## Abstract

Parkinson’s disease (PD) is a long term disorder affects the central nervous system and we aim to determine the relative efficacy of the current available drugs used in PD. Firstly, we performed a systematic review in current literature and eligible studies were retrieved from online databases, relevant data were extracted. Efficacy of these medications was assessed by different Unified Parkinson’s Disease Rating Scales (UPDRS). Mean difference (MD) and odds ratio (OR) were produced by pairwise or network meta-analysis (NMA). Finally, we performed a cluster analysis for the included medications with respect to their surface under the cumulative ranking curve (SUCRA). Pairwise meta-analysis suggests that selegiline had a higher ranking in UPDRS II, UPDRS III and UPDRS total than bromocriptine and levodopa. Selegiline was more tolerable than bromocriptine (OR = 0.62, CI: 0.39 to 0.98) and pramipexole was less tolerable than levodopa (OR = 1.43, CI = 1.00 to 2.04). Results of NMA indicate that patients with levodopa, pramipexole, ropinirole and selegiline exhibited a significantly improved UPDRS III than those with lazabemide. To sum up, levodopa, selegiline, ropinirole and rotigotine were recommended for PD patients as they appeared relatively high efficacy and tolerability.

Parkinson’s disease (PD) is a chronic neurodegenerative disorder accompanied with several cardinal motor characteristic symptoms including resting tremor, postural instability, rigidity and bradykinesia. Previous studies have proved that PD was a result of the depletion of dopaminergic neurons in substantianigra^1^. In addition, age *per se* is a key factor that affects PD’s pathogenesis and progression by changing cellular processes and functions that related with neurodegeneration^2^.

PD is one of the most common neurodegenerative diseases ranking only second to Alzheimer’s disease^3^. According to a rough estimation performed by Twelves *et al*., incidence of PD around the world was around 16–19 per 100,000 people per year, with highest incidence in males aging between 70 and 79 years^4^. Since population worldwide is aging gradually, it is predicted by Dorsey *et al*. that the population of patients affected by PD will double in 2030^5^. Apart from the high prevalence rate among elder males, PD is also characterized with a high mortality. In a 20 years follow-up of 136 patients diagnosed with new-onset PD, a high mortality of 74% was observed and dementia occurred in 83% of the remaining survivors^6^.

So far, all treatments for PD were aimed at alleviating its clinical symptoms and improving the life quality of patients and no curative therapy has been developed to reverse the underlying neurodegenerative process^7^. Levodopa, dopamine agonists (DA) and monoamine oxidase type B inhibitors (MAOBI) are mainstream drugs that are widely used as first-line treatments of PD. Among them, levodopa performed best in symptomatic control and it guaranteed at least 50% improvement in symptomatic for a period of 2 to 3 years^8^. However, levodopa would cause increased dyskinesia, motor fluctuations and other adverse effects in the long term^9^,^10^. To prolong the beneficial effect of motor symptomatic control, levodopa is often combined with DA or MAOBI as adjunctive therapy for all-stage PD patients. DA, such as bromocriptine, cabergoline, pergolide, pramipexole, ropinirole and rotigotine, is a class of drugs that act on D2 receptors and work well in controlling motor fluctuations^7^. Apart from acting as an adjunct therapy to levodopa, it’s also widely used as monotherapy for PD in early stages to delay the utilization of levodopa therapy^11^. Monoamine oxidase type B (MAOB) is the leading enzyme regulating concentrations of neurotransmitters such as acetylcholine and dopamine that are related with emotion, movement and cognition in human brain^12^. In clinical trials, its inhibitor has been used to down-regulate the degree of on-off motor fluctuations^13^. Rasagiline and selegiline are both selective and irreversible MAOBI and now used as anti-Parkinson drug or adjunct to levodopa, wherein former one is more potent *in vivo*^13^,^14^. Besides, lazabemide is also a drug of MAOBI. Similarly, their adverse motor effects such as dizziness, wearing-off, on-off phenomena and insomnia also raised concerns.

Tough numerous placebo-controlled trails have been implemented to assess efficacy of anti-Parkinson drugs, no comprehensive comparisons for efficacy and tolerability among all available treatments were conducted. As such, present study was designed to make comparisons of monotherapy’s efficacy and tolerability among ten drugs mentioned above by combining evidence from previous randomized controlled trials (RCTs).

## Results

### Study characteristics

**Figure 1:**
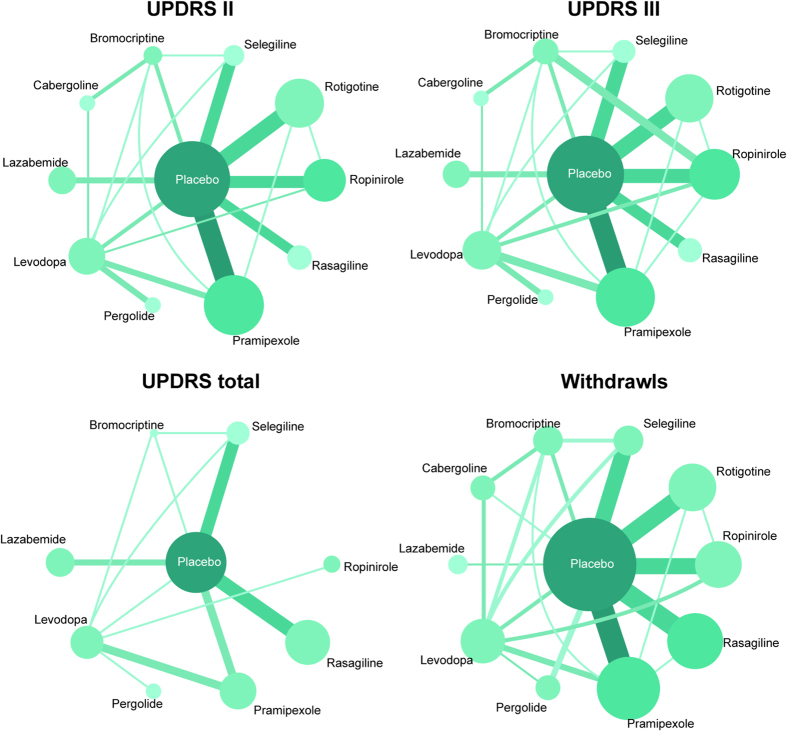
The network plot of included trials. Each node represents a therapy of PD, the number beside the nodes represents the number of people involved and the number between two nodes represents the number of study involved in the head-to-head comparison.

As presented in Figure S1, 110 publications involving 24,864 participants were finally included in the present study after screening 1,154 publications according to the inclusion criteria^13^,^15^,^16^,^17^,^18^,^19^,^20^,^21^,^22^,^23^,^24^,^25^,^26^,^27^,^28^,^29^,^30^,^31^,^32^,^33^,^34^,^35^,^36^,^37^,^38^,^39^,^40^,^41^,^42^,^43^,^44^,^45^,^46^,^47^,^48^,^49^,^50^,^51^,^52^,^53^,^54^,^55^,^56^,^57^,^58^,^59^,^60^,^61^,^62^,^63^,^64^,^65^,^66^,^67^,^68^,^69^,^70^,^71^,^72^,^73^,^74^,^75^,^76^,^77^,^78^,^79^,^80^,^81^,^82^,^83^,^84^,^85^,^86^,^87^,^88^,^89^,^90^,^91^,^92^,^93^,^94^,^95^,^96^,^97^,^98^,^99^,^100^,^101^,^102^,^103^,^104^,^105^,^106^,^107^,^108^,^109^,^110^,^111^,^112^,^113^,^114^,^115^,^116^,^117^,^118^,^119^,^120^,^121^,^122^,^123^. Baseline characteristics were shown in Table 1. As we can see, all these studies were designed as RCTs and most of them were double-blind RCTs. Patients were diagnosed as either early or advanced PD and most of them were male above 60. Besides, we draw a network of included trials in Fig. 1, from which we observed that most RCTs had taken placebo as the control group. Among all interventions, Pramipexole, Ropinirole, Levodopa and Rasagiline were involved in most studies and had relative bigger sample sizes.

### Meta-analysis results for pair-wise comparisons

Meta-analysis results for pair-wise comparisons were shown in Table 2. We found that lazabemide exhibited a worse efficacy with respect to UPDRS II compared with placebo (MD = 0.82, CI: 0.29 to 1.34). Patients with levodopa, pramipexole, ropinirole, rotigotine and selegiline all functioned better with respect to UPDRS II and III than those with placebo. With respect to UPDRS total, lazabemide also functioned worse than placebo (MD = 1.88, CI: 0.57 to 3.19) while bromocriptine, levodopa, rasagiline and selegiline functioned better. Besides, selegiline had a higher score in UPDRS II, UPDRS III and UPDRS total than bromocriptine and levodopa. As for withdrawal, only rotigotine had a significant lower withdrawal rate than placebo. Besides, selegiline was more tolerable than bromocriptine (OR = 0.62, CI: 0.39 to 0.98) and pramipexole had a higher withdraw rate than levodopa (OR = 1.43, CI = 1.00 to 2.04).

### Network meta-analysis results

As we can see in Table 3 and Figures S2–5, for UPDRS II, levodopa, pramipexole, ropinirole, rotigotine and selegiline exhibited increased efficacy compared to placebo and lazabemide All interventions except for cabergoline, lazabemide, pergolide and rasagiline exhibited an increased efficacy compared to the placebo with respect to UPDRS III. Patients with levodopa, pramipexole, ropinirole and selegiline exhibited a significantly improved UPDRS III than those with lazabemide. Our NMA suggests that only patients with selegiline exhibited significantly improved UPDRS total than those with placebo (MD = −6.04, CrI: −11.07 to −0.83). On the other hand, patients with levodopa or ropinirole exhibited a lower risk of withdrawals compared to those with placebo and bromocriptine (ORs < 1). Finally, selegiline appeared to have higher withdraw rate than levodopa, ropinirole and rotigotine with respect to the likelihood of withdrawals (OR = 2.43, CrI: 1.42 to 4.23; OR = 2.17, CrI: 1.27 to 3.84; OR = 1.93, CrI: 1.08 to 3.51).

### Cumulative ranking probability as a ranking scheme

**Figure 2:**
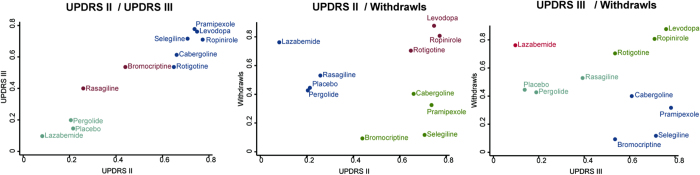
Clustered ranking plot of the network. The plot is based on cluster analysis of surface under the cumulative ranking curves (SUCRA) values. Each plot shows SUCRA values for two outcomes. Each color represents a group of treatments that belong to the same cluster. Treatments lying in the upper right corner are more effective and safe than the other treatments.

Table 4 and Figure S6 showed the cumulative ranking probability of all interventions based on each outcome. Three drugs including ropinirole, pramipexole, and selegiline ranked first in UPDRS II, III and total (with the value of 0.773, 0.777 and 0.918 respectively, and levodopa had the highest rank in withdraw rate. Besides, selegiline ranked the first in UPDRS total but the last in withdrawal. Levodopa and ropinirole had a high ranking when taking withdrawals into consideration. Besides, lazabemide was a mild intervention with both low efficacy rank and withdrawal rate. Cluster analysis presented results above in a more intuitional way (Fig. 2). Interventions with the same level of SUCRA values are displayed in the same color. Levodopa, ropinirole and rotigotine fall into the group with both the most favorable SUCRA values and tolerability as well.

### Consistency

**Figure 3:**
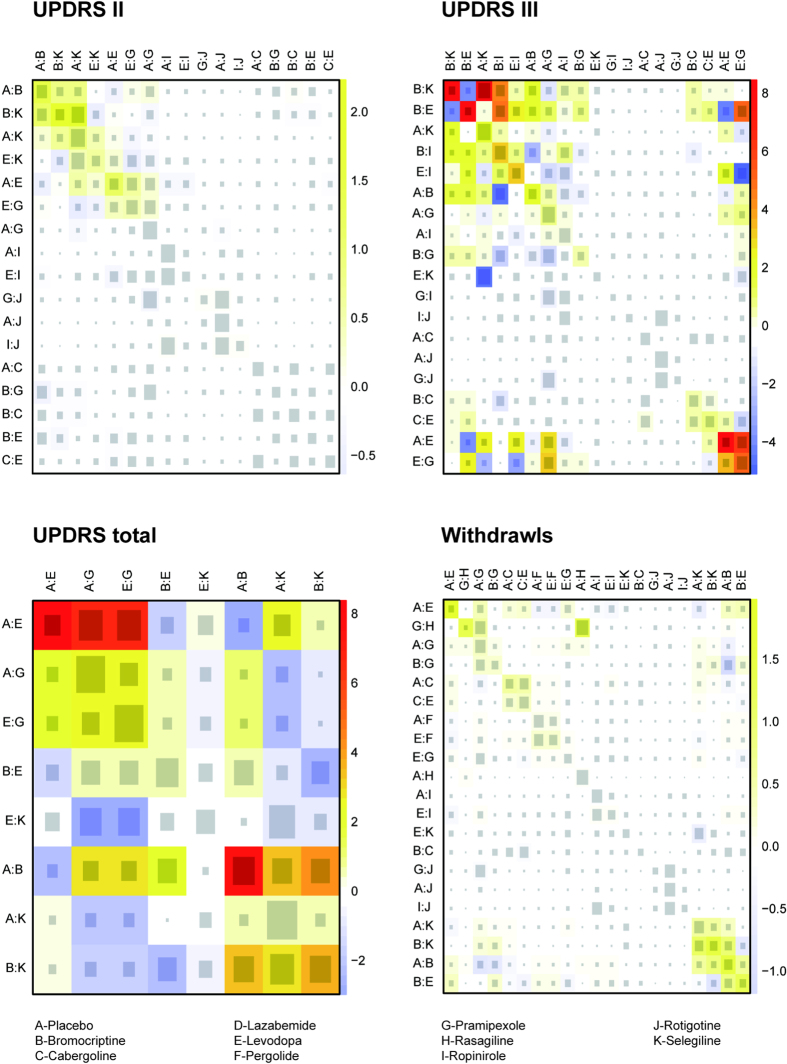
Net heat plot. The size of the gray squares indicates the contribution of the direct evidence (shown in the column) to the network evidence (shown in the row). The colors are associated with the change in inconsistency between direct and indirect evidence (shown in the row). Blue colors indicate an increase of inconsistency and warm colors indicate a decrease.

In node-splitting plot (Figure S7), all *P*-values are higher than 0.05, which indicated a relatively satisfactory consistency between direct and indirect evidence. In heat map (Fig. 3), consistency between direct evidence and NMA results in UPDRS II and withdrawal was well-pleasing. However, there appeared to be some significant inconsistency between direct and indirect evidence in UPDRS III and UPDRS total.

## Discussion

This study made a comprehensive comparison for the tolerability and efficacy among anti-Parkinson drugs by using a network meta-analysis. Interventions were grouped into placebo, DA (Pramipexole, Ropinirole and Rotigotine), MAOBI (- Rasagiline and Selegiline) and Levodopa. Efficacy outcomes included unified PD rating scale (UPDRS) II, UPDRS III and UPDRS total. Taking tolerability, efficacy and adverse effect into consideration, we also examined withdraw rate, treatments with high withdraw rate means lacking of efficacy, safety or easy to become tolerant. To our knowledge, this is the first study that well explored the efficacy and tolerability ranking of these three types of drugs for Parkinson with a great range of outcomes included.

Levodopa is an intervention that is widely used in clinical trials with good control of symptoms of PD. Noticeably, levodopa is one of the best tolerated treatments for PD, particularly in the elderly patients^124^. Our research indicated the same result that levodopa ranked high in UPDRS II and III, and maintained a very low withdraw rate, which possessed a very favorable balance between efficacy and tolerability and worthy of recommendation. However, it may still cause several long-term adverse events including motor complications and dyskinesia^125^.

The efficiency of DA in reducing motor fluctuations and dyskinesias has been reported by previous studies^126^,^127^. For instance, Rascol *et al*. found that patients with early PD can be well controlled with a low risk of dyskinesia by an initial therapy of ropinirole, an agent of DA, alone. Also, a levodopa-controlled trial conducted by FulvioBracco *et al*. suggested that patients with PD were in a lower risk of motor fluctuations when treated with cabergoline, another agent of DA, though the relative safety was at the expense of a mildly improved clinical symptom^24^. Thus, these drugs were usually added into levodopa to weaken its adverse effects in clinical trials.

Compared to levodopa, MAOBI was found to decrease the incidence of disability during the treatment and motor fluctuations without any notable mortality rate or adverse effects^128^. Whereas, this meta-analysis conducted by Ives N.J. *et al*. was short of direct comparisons between MAOBIs and other types of anti-Parkinson drugs and thus was not sufficient.

Though our results were consistent with most previous trials, there still exist several flaws. One of the limitations in this study is that we only research on the monotherapy for PD. However, in clinical trials, it’s common that these drugs were applied together to offset the corresponding adverse effects or the low efficacy rate raised by monotherapy. Besides, some other influence factors such as dosages, design and sample size may affect the accuracy and reliability of our results. For this, more clinical trials in comparisons of these interventions are in desperate need.

In our results, according to SUCRA, four drugs including levodopa, pramipexole, ropinirole and selegiline all had a well performance in UPDRS II and UPDRS III. And among them, selegiline had a highest UPDRS total and highest withdraw rate. Levodopa and ropinirole had a higher ranking when withdrawals were taken into consideration. Although lazabemide was a mild intervention with low efficacy rank and withdrawal rate, it has not been introduced in the market and not available for the patients. Besides, in cluster analysis, levodopa, ropinirole and rotigotine steadily ranked first in view of three endpoints including UPDRS II, UPDRS III and withdrawal. The network meta-analysis integrated evidence from 110 independent RCTs and thus provided an accurate results and smaller random errors.

In conclusion, levodopa, selegiline, ropinirole and rotigotine were recommended for PD patients for their relatively high efficacy and tolerability. If necessary, an appropriate composition of these drugs will perform well with a relative low risk of adverse effects and a high efficacy.

## Methods and Materials

### Search strategy

Publications in PubMed, Embase and Cochrane Library were retrieved without language restrictions. Keywords included Parkinson disease, bromocriptine, cabergoline, lazabemide, levodopa, pergolide, pramipexole, rasagiline, ropinirole, rotigotine, selegiline and RCTs. Publications were first screened by reviewing their titles and abstracts and further reviewed by scanning full texts. In addition, cited references attached to the included documents were also retrieved.

### Inclusion criteria

Studies were included when they met the following criteria:

1. Experiments were designed as RCTs comparing the efficacy of treatments for PD.
2. Patients or participants were adults diagnosed with PD.
3. Outcomes in studies included at least one of the following endpoints: Unified Parkinson’s Disease Rating Scale (UPDRS) II, UPDRS III, UPDRS total and withdrawals.
4. Interventions included at least one of the following drugs: bromocriptine, cabergoline, lazabemide, levodopa, pergolide, pramipexole, rasagiline, ropinirole, rotigotine and selegiline.

### Data extraction

After reading through the full text, the following information was extracted from each independent study: author, publication year, sample size, gender ratio, design, blind, follow-up, age, condition of PD, intervention and dosage. As for outcomes, several unified PD rating scales (UPDRS) including UPDRS II, UPDRS III and UPDRS total were extracted if available, which has been considered as the primary efficacy outcomes in this analysis; meanwhile the rate of withdraw during the treatment was adopted as an endpoint integrating tolerability, efficacy and adverse effect as a whole.

### Statistical analysis

STATA version 12.0 (Stata Corp, College Station, TX, USA) software was applied in traditional meta-analysis. Firstly, the heterogeneity was examined by using Cochran’s *Q*-statistic or *I*^*2*^ test. When significant heterogeneity did not exist (*P* > 0.05 or *I*^*2*^<50%), a fixed-effects model (*Mantel-Haenszel* method) was performed. Otherwise, we tried to find out the source of heterogeneity and eliminate the potential source of heterogeneity. Alternatively, a random-effects model (*Der Simonian-Laird* method) would be applied. For count data such as withdrawal, odd ratios (OR) and corresponding 95% confidence interval (CI) were calculated. For measurement data including UPDRS II, UPDRS III, UPDRS total, withdrawals, the mean difference (MD) and the corresponding 95% CI were calculated.

WinBUGS (MRC Bio-statistics Unit, Cambridge, UK) software was applied in network meta-analysis (NMA). To combine both direct and indirect evidence, a *Markov chain Monte Carlo* method and Bayesian networks were built. Similar with cases in traditional meta-analysis, OR and MD were separately used in count data and measurement data. Meanwhile, the corresponding 95% credential interval (CrI) was also calculated. To illustrate the results from NMA more directly, the surface under the cumulative ranking curve (SUCRA) was drawn and presented the ranking according to different endpoints. SUCRA enable us to identify the best treatment overall. The value of SUCRA would be 1 (i.e. 100%) for the best and 0 for the worst. In addition, a cluster analysis was conducted to combine the ranking under two independent endpoints and divide the interventions into several levels in view of their performan

Consistency between direct and indirect evidence was assessed by *P*-value and *P* > 0.05 exhibited a significant consistency. A heat map was plotted to present the consistency between direct evidence and NMA results, in which red indicates significant inconsistency while blue indicates significant consistency.

## Additional Information

**How to cite this article:** Zhuo, C. *et al*. Comparison for Efficacy and Tolerability among Ten Drugs for Treatment of Parkinson’s Disease: A Network Meta-Analysis. *Sci. Rep.* **7**, 45865; doi: 10.1038/srep45865 (2017).

**Publisher's note:** Springer Nature remains neutral with regard to jurisdictional claims in published maps and institutional affiliations.

## Supplementary Material

Supplementary Information

## Figures and Tables

**Table 1:** Characteristics of studies included in the network meta-analysis.

Study	Size	Male	Blind*	Follow-up (months)	Age>	Early/Advanced PD	Intervention	Dosage
Adler 1997	241	62.2%	2	6	62.8	Early	Ropinirole vs. Placebo	15.7 mg/d
Ahlskog 1988	49	71.4%	2	6	50.0	Advanced	Pergolide vs. Placebo	0.75 mg/d
Ahlskog 1996	27	74.1%	2	6	63.9	Early/Advanced	Cabergoline vs. Placebo	5 mg/d
Allain 1993	93	53.8%	2	3	65.0	Early	Selegiline vs. Placebo	10 mg/d
Antonini 2015	349	56.2%	2	4.8	67.5	Early/Advanced	Rotigotine vs. Placebo	12 mg/d
Barone 2007	624	62.0%	2	10	64.6	Advanced	Ropinirole vs. Placebo	18 mg/d
Barone 2010	296	47.3%	2	3	67.0	Early	Pramipexole vs. Placebo	2.18 mg/d
Barone 2015	123	52.8%	2	3	66.0	Early	Rasagiline vs. Placebo	1 mg/d
Blindeauer 2003	242	63.6%	2	2.8	61.3	Early	Rotigotine vs. Placebo	4.5, 9, 13.5, 18 mg
Bracco 2004	419	51.0%	2	60	61.4	Early	Cabergoline vs. Levodopa	2.85 mg/d vs 784 mg/d
Brooks 1998	63	51.0%	2	3	58.3	Early/Advanced	Ropinirole vs. Placebo	6.54 mg/d
Brunt 2002	206	59.6%	2	6	65.8	Advanced	Ropinirole vs. Bromocriptine	9, 10, 14 mg/d vs 18, 19, 24 mg/d
Caraceni 2001	473	52.0%	0	34	63.3	Early/Advanced	Levodopa vs. Bromocriptine vs. Selegiline	750 mg/d vs 60 mgd vs 10 mg/d
Giladi 2007	561	57.7%	2	9.3	61.2	Early	Rotigotine vs. Ropinirole vs. Placebo	8 mg/d vs 14.1 mg/d
Golbe 1988	96	—	2	1.5	62.4	Advanced	Selegiline vs. Placebo	10 mg/d
Grosset 2005	106	67.0%	2	17.3	61.0	Early	Pergolide vs. Placebo	0.05 mg/d
Guttman 1997	246	63.4%	2	9	62.7	Advanced	Pramipexole vs. Bromocriptine vs. Placebo	3.36 mg/d vs 22.64 mg/d
Hanagasi 2011	48	68.8%	2	3	66.4	Early	Rasagiline vs. Placebo	1 mg/d
Hauser 2007	69	58.0%	2	120	62.1	Early	Ropinirole vs. Levodopa	14.5 mg/d vs 800.2 mg/d
Hauser 2010	259	55.6%	2	4.5	62.1	Early	Pramipexole vs. Placebo	1.37, 1.39 mg/d
Hauser 2014	326	68.0%	2	4.5	62.6	Early	Rasagiline vs. Placebo	1 mg/d
Hauser 2015	778	56.0%	2	3	63.3	Advanced	Rasagiline vs. Placebo	1 mg/d
Hely 1994	126	55.6%	2	60	62.0	Early	Bromocriptine vs. Levodopa	31 mg/d vs 427 mg/d
Holloway 2000	301	64.8%	2	23.5	61.2	Early	Pramipexole vs. Levodopa	1.5 mg/d vs 300 mg/d
Holloway 2004	183	64.5%	2	48	60.9	Early	Pramipexole vs. Levodopa	1.5 mg/d vs 300 mg/d
Holloway 2009	301	61.7%	0	72	60.2	Early	Pramipexole vs. Levodopa	3 mg/d vs 450 mg/d
Hubble 1995	55	63.6%	2	2.25	63.3	Early	Pramipexole vs. Placebo	0.3–4.5 mg/d
Hutton 1996	188	66.5%	2	6	63.4	Early/Advanced	Cabergoline vs. Placebo	0.5–5 mg/d
Im 2003	76	54.0%	0	4	61.7	Early/Advanced	Ropinirole vs. Bromocriptine	7.9 mg/d vs 15.4 mg/d
Inzelberg 1996	44	63.6%	2	9	71.0	Early/Advanced	Cabergoline vs. Bromocriptine	3.18 mg/d vs 22.05 mg/d
Jankovic 2014	883	—	2	9	62.8	Early	Rasagiline vs. Placebo	1 mg/d
Jansen 1978	23	56.5%	2	5	59.0	Advanced	Bromocriptine vs. Placebo	71 mg/d
Kieburtz 1993	201	67.7%	2	1	63.0	Early	Lazabemide vs. Placebo	100, 200, 400 mg/d
Kieburtz 1996	321	71.2%	2	13	64.1	Early	Lazabemide vs. Placebo	23, 50, 100, 200 mg/d
Kieburtz 1997	264	64.4%	2	2.5	61.7	Early	Pramipexole vs. Placebo	1.5, 3, 4.5, 6 mg/d
Kieburtz 2011	311	66.6%	2	3	62.8	Early	Pramipexole vs. Placebo	0.50 mg tid, 0.50, 0.75 mg bid
Kim 2015	48	50.0%	2	0.2	24.0	Healthy	Rotigotine vs. Placebo	2&4 mg/d
Koller 1993	376	—	2	3	—	Early	Selegiline vs. Placebo	10 mg/d
Kulisevsky 1998	20	35.0%	0	6	65.7	Early/Advanced	Pergolide vs. Levodopa	2.8 mg/d vs 435 mg/d
Kulisevsky 2000	20	35.0%	0	6	65.7	Early	Pergolide vs. Levodopa	2.8 mg/d vs 435 mg/d
Larsen 1999	163	—	2	60	55.0	Early	Selegiline vs. Placebo	10 mg/d
LeWitt 2007	349	63.9%	2	7.5	65.0	Advanced	Rotigotine vs. Placebo	7.16, 9.51 mg/d
Lieberman 1997	360	65.0%	2	8	63.3	Advanced	Pramipexole vs. Placebo	2.44 mg/d
Lieberman 1998	149	—	2	6	—	Early/Advanced	Ropinirole vs. Placebo	15.75 mg/d
Lim 2015	30	53.3%	2	3	67.2	Advanced	Rasagiline vs. Placebo	1 mg/d
Mally 1995	20	65.0%	2	1.5	62.5	Early/Advanced	Selegiline vs. Placebo	10 mg/d
Marek 2002	82	62.6%	2	46	61.0	Early	Pramipexole vs. Levodopa	1.5 mg/d vs 300 mg/d
Maier Hoehn 1985	36	75.0%	2	10	62.9	Early/Advanced	Bromocriptine vs. Placebo	1.25–20 mg/d
Mendzelevski 2014	247	46.8%	2	0.3	21.0	Healthy	Rasagiline vs. Placebo	1, 2, 6 mg/d
Mizuno 2003	315	52.7%	2	3	64.6	Advanced	Pramipexole vs. Bromocriptine vs. Placebo	3.24 mg/d vs 17.75 mg/d
Mizuno 2007	241	44.4%	2	4	65.0	Advanced	Ropinirole vs. Placebo	7.12 mg/d
Mizuno 2013	176	39.8%	2	3.8	66.0	Early	Rotigotine vs. Placebo	12.8 mg/d
Mizuno 2014	420	41.3%	2	5	65.0	Advanced	Rotigotine vs. Ropinirole vs. Placebo	12.9 mg/d vs 9.2 mg/d
Moller 2005	354	65.0%	2	7.8	64.0	Advanced	Pramipexole vs. Placebo	3.7 mg/d
Myllyla 1995	44	47.7%	2	24	60.7	Early/Advanced	Selegiline vs. Placebo	10 mg/d
Myllyla 1997	44	48.8%	2	60	60.7	Early/Advanced	Selegiline vs. Placebo	10 mg/d
Navan 2003	10	60.0%	2	4 h	65.3	Early	Pramipexole vs. Pergolide vs. Placebo	0.5 mg vs 0.5 mg
Navan 2003	30	63.3%	2	3	69.0	Early/Advanced	Pramipexole vs. Pergolide vs. Placebo	4.5 mg/d vs 4.5 mg/d
Nicholas 2014	514	69.8%	2	4	64.5	Advanced	Rotigotine vs. Placebo	2, 4, 6, 8 mg/d
Nomoto 2014	174	44.8%	2	4.8	67.0	Advanced	Rotigotine vs. Placebo	16 mg/d
Oertel 2006	294	56.8%	2	36	58.9	Early	Pergolide vs. Levodopa	3.23 mg/d vs 504 mg/d
Olanow 1994	376	63.6%	2	6	63.0	Advanced	Pergolide vs. Placebo	2.94 mg/d
Olanow 1995	101	68.3%	2	14	66.2	Early	Selegiline vs. Levodopa vs. Bromocriptine vs. Placebo	10 mg/d vs 400 mg/d vs 28 mg/d
Olanow 2009	1176	61.1%	2	9	62.2	Early/Advanced	Rasagiline vs. Placebo	1, 2 mg/d
Pahwa 2007	393	62.9%	2	6	66.2	Advanced	Ropinirole vs. Placebo	18.8 mg/d
Pahwa 2014	381	55.6%	2	7.5	65.0	Early	Levodopa vs. Placebo	145, 245, 390 mg tid
Palhagen 1998	157	59.3%	2	6	63.7	Early	Selegiline vs. Placebo	10 mg/d
Parkinson Study Group 1994	137	66.4%	2	1	67.0	Early	Lazabemide vs. Placebo	100, 200, 400 mg/d
Pinter 1999	78	65.4%	2	2.8	60.1	Advanced	Pramipexole vs. Placebo	3.59 mg/d
Poewe 2007	506	62.9%	2	6.8	64.0	Advanced	Pramipexole vs. Rotigotine vs. Placebo	3.1 mg/d vs 12.95 mg/d
Poewe 2011	539	55.5%	2	8.3	62.0	Early	Pramipexole vs. Placebo	2.9 mg/d
Poewe 2015	174	57.5%	2	12	65.0	Early	Rasagiline vs. Placebo	1 mg/d
Pogarell 2002	84	72.3%	2	3	63.6	Early/Advanced	Pramipexole vs. Placebo	4.1 mg/d
Presthus 1983	38	52.6%	2	1	65.8	Early/Advanced	Selegiline vs. Placebo	5 mg/d
Rabey 2000	70	55.7%	2	3	57.0	Early/Advanced	Rasagiline vs. Placebo	0.5, 1, 2 mg/d
Rascol 1996	46	60.9%	2	3	62.5	Early/Advanced	Ropinirole vs. Placebo	3.3 mg/d
Rascol 1998	268	61.2%	2	6	63.0	Early	Ropinirole vs. Levodopa	9.7 mg/d vs 464.0 mg/d
Rascol 2000	268	61.6%	2	60	63.0	Early	Ropinirole vs. Levodopa	16.5 mg/d vs 753 mg/d
Rascol 2005	460	62.2%	2	4.5	64.3	Early/Advanced	Rasagiline vs. Placebo	1 mg/d
Rascol 2015	68	52.9%	2	3	65.9	Advanced	Rotigotine vs. Placebo	14.7 mg/d
Rektorova 2003	41	61.0%	2	8	61.5	Advanced	Pramipexole vs. Pergolide	2.7 mg/d vs 3.0 mg/d
Rinne 1998	412	48.5%	2	6	61.5	Early	Cabergoline vs. Levodopa	3 mg/d vs 500 mg/d
Sampaio 2011	225	58.2%	2	6	61.8	Early	Pramipexole vs. Placebo	2.25 mg/d
Schapira 2011	507	54.9%	2	4.5	61.5	Advanced	Pramipexole vs. Placebo	2.7, 2.8 mg/d
Schiwid 2005	472	64.6%	2	6.5	63.3	Early	Rasagiline vs. Placebo	0.5, 1.0 mg/d
Sethi 1998	147	62.6%	2	12	62.0	Early	Ropinirole vs. Placebo	17.9 mg/d
Shannon 1997	335	60.6%	2	6	62.7	Early	Pramipexole vs. Placebo	3.8 mg/d
Siderowf 2002	404	63.6%	2	6.5	60.8	Early	Rasagiline vs. Placebo	1, 2 mg/d
Singer 2007	405	62.0%	2	10	65.0	Early	Ropinirole vs. Placebo	12.4 mg/d
Smith 2015	191	—	2	9	61.2	Early	Rasagiline vs. Placebo	1–2 mg/d
Steiger 1996	37	—	2	3	62.1	Early/Advanced	Cabergoline vs. Placebo	5.4 mg/d
Stern 2004	56	67.9%	2	2.5	61.5	Early	Rasagiline vs. Placebo	1, 2, 4 mg/d
Stocchi 2008	161	54.0%	2	5	60.3	Early	Ropinirole vs. Ropinirole	8.9 mg/d vs 18.6 mg/d
Stocchi 2011	69	69.6%	2	4.5	64.2	Early/Advanced	Rasagiline vs. Placebo	1 mg/d
Storch 2013	35	68.6%	1	3	61.7	Early	Cabergoline vs. Levodopa	3 mg/d vs 300 mg/d
Tanner 2007	144	65.0%	2	2.5	65.0	Advanced	Pramipexole vs. Placebo	4.5 mg/d
Tetrud 1989	54	68.5%	2	36	61.0	Early	Selegiline vs. Placebo	10 mg/d
Thomas 2006	52	55.8%	2	24	56.2	Early	Ropinirole vs. Pramipexole	15 mg/d vs 521 mg/d
Timmermann 2015	346	61.7%	2	6	67.0	Early	Rotigotine vs. Placebo	8.5 m/d
Toyokur 1985	222	49.1%	2	2	63.0	Early/Advanced	Bromocriptine vs. Placebo	2.5 mg/d
Trenkwalder 2011	287	64.1%	2	2	64.7	Early/Advanced	Rotigotine vs. Placebo	16 mg/d
Utsumi 2012	91	47.3%	0	60	62.0	Early	Cabergoline vs. Levodopa	2.9 mg/d vs 325 mg/d
Viallet 2013	109	62.4%	2	3.8	62.6	Early	Rasagiline vs. Pramipexole	1 mg/d vs 1.5 mg/d
Waters 2004	140	63.6%	2	3	65.3	Early/Advanced	Selegiline vs. Placebo	1.875 mg/d
Weintraub 2016	170	78.0%	2	6	67.5	Early	Rasagiline vs. Placebo	1 mg/d
Wermuth 1998	69	58.0%	2	2.8	62.1	Advanced	Pramipexole vs. Placebo	5 mg/d
Whone 2003	162	67.3%	2	24	60.5	Early	Ropinirole vs. Levodopa	12.2 mg/d vs 558.7 mg/d
Wong 2003	150	69.3%	2	3.8	60.0	Early/Advanced	Pramipexole vs. Placebo	2.44 mg/d
Zhang 2013	219	59.8%	2	3	61.6	Early/Advanced	Rasagiline vs. Placebo	1 mg/d
Zhang 2014	345	64.1%	2	6	63.9	Advanced	Ropinirole vs. Placebo	11.4 mg/d

^*^Blind: 0, open label; 1, single blind; 2, double blind. Abbreviation: PD, Parkinson’s disease

**Table 2:** Meta-analysis results for pair-wise comparisons according to UPDRS II, UPDRS III, UPDRS total represented by mean difference (MD) and 95% confidence interval (CI) and withdrawals represented by odds ratio (OR) with 95% confidence interval (CI).

Treatment 1	Treatment 2	UPDRS II	UPDRS III	UPDRS total	Withdrawals
Bromocriptine	Placebo	**−1.02 (−1.31, −0.73)**	−2.27 (−6.11, 1.56)	**−1.30 (−1.87, −0.73)**	1.03 (0.57, 1.85)
Cabergoline	Placebo	**−2.30 (−4.40, −0.20)**	−1.60 (−4.07, 0.87)	—	0.65 (0.31, 1.41)
Lazabemide	Placebo	**0.82 (0.29, 1.34)**	0.83 (−0.18, 1.83)	**1.88 (0.57, 3.19)**	0.68 (0.34, 1.36)
Levodopa	Placebo	**−2.26 (−4.49, −0.03)**	**−6.05 (−12.06, −0.04)**	**−4.10 (−4.75, −3.45)**	0.98 (0.58, 1.63)
Pergolide	Placebo	—	—	—	1.02 (0.66, 1.58)
Pramipexole	Placebo	**−1.48 (−2.02, −0.94)**	**−3.86 (−5.87, −1.86)**	1.25 (−7.66, 10.15)	0.96 (0.73, 1.27)
Rasagiline	Placebo	−0.17 (−1.94, 1.60)	−1.86 (−4.30, 0.58)	**−2.30 (−4.00, −0.60)**	0.94 (0.76, 1.15)
Ropinirole	Placebo	**−1.90 (−2.28, −1.52)**	**−5.05 (−5.95, −4.15)**	—	0.76 (0.56, 1.05)
Rotigotine	Placebo	**−1.43 (−2.50, −0.36)**	**−3.12 (−5.48, −0.76)**	—	**0.78 (0.62, 0.98)**
Selegiline	Placebo	**−1.46 (−2.48, −0.43)**	**−3.85 (−5.89, −1.81)**	**−6.46 (−10.50, −2.43)**	1.39 (0.94, 2.04)
Cabergoline	Bromocriptine	0.00 (−3.04, 3.04)	2.00 (−5.68, 9.68)	—	0.71 (0.20, 2.60)
Levodopa	Bromocriptine	−0.20 (−0.53, 0.13)	**−2.60 (−2.95, −2.25)**	**−2.80 (−3.41, −2.19)**	0.37 (0.09, 1.50)
Pramipexole	Bromocriptine	−0.73 (−1.79, 0.33)	−1.77 (−4.54, 1.00)	—	0.92 (0.54, 1.58)
Ropinirole	Bromocriptine	—	0.06 (−0.77, 0.89)	—	
Selegiline	Bromocriptine	**−1.00 (−1.28, −0.72)**	**−3.00 (−3.34, −2.66)**	**−4.10 (−4.64, −3.56)**	**0.62 (0.39, 0.98)**
Levodopa	Cabergoline	−1.30 (−3.32, 0.72)	−0.70 (−5.86, 4.46)	—	0.72 (0.47, 1.11)
Pergolide	Levodopa	1.88 (−0.34, 4.11)	**5.10 (3.33, 6.87)**	**8.50 (5.53, 11.47)**	1.17 (0.76, 1.79)
Pramipexole	Levodopa	**−0.88 (−1.56, −0.19)**	−1.93 (−4.82, 0.96)	−2.12 (−5.51, 1.27)	**1.43 (1.00, 2.04)**
Ropinirole	Levodopa	0.60 (−0.20, 1.40)	1.50 (−7.85, 10.84)	**2.60 (0.03, 5.17)**	1.04 (0.72, 1.50)
Selegiline	Levodopa	**−0.80 (−1.15, −0.45)**	**−0.40 (−0.74, −0.06)**	**−1.30 (−1.93, −0.67)**	1.96 (0.67, 5.75)
Rasagiline	Pramipexole	—	—	—	0.40 (0.10, 1.57)
Ropinirole	Pramipexole	0.40 (−0.47, 1.27)	0.20 (−1.94, 2.34)	—	0.76 (0.42, 1.35)
Rotigotine	Pramipexole	—	1.60 (−0.03, 3.23)	—	—
Rotigotine	Ropinirole	−0.60 (−1.63, 0.43)	−1.40 (−3.21, 0.41)	—	1.14 (0.63, 2.09)

Abbreviation: UPDRS, Unified Parkinson’s Disease Rating Scale.

**Table 3:** Network meta-analysis results for UPDRS II, UPDRS III, UPDRS total represented by mean difference (MD) and 95% credible interval (CrI), withdrawals represented by odds ratio (OR) and 95% CrI. In lower half of the table, row treatments are compared against column treatments, whereas in the upper half, column treatments are compared against row treatments.

UPDRS III												
	Treatment	Placebo	Bromocriptine	Cabergoline	Lazabemide	Levodopa	Pergolide	Pramipexole	Rasagiline	Ropinirole	Rotigotine	Selegiline
	Placebo	Placebo	−3.18 (−5.91, −0.44)	−3.64 (−8.70, 1.33)	0.85 (−3.04, 4.56)	−4.33 (−6.85, −1.83)	0.11 (−5.15, 5.19)	−4.38 (−6.09, −2.67)	−2.06 (−5.06, 0.79)	−4.05 (−6.08, −2.04)	−3.09 (−5.37, −0.79)	−4.15 (−6.63, −1.70)
	Bromocriptine	−0.74 (−2.26, 0.78)	Bromocriptine	−0.47 (−5.13, 4.17)	4.04 (−0.78, 8.66)	−1.16 (−4.35, 1.98)	3.30 (−2.39, 8.82)	−1.19 (−4.20, 1.74)	1.10 (−2.91, 5.08)	−0.87 (−3.41, 1.64)	0.13 (−3.49, 3.57)	−0.97 (−4.49, 2.54)
	Cabergoline	−1.42 (−3.74, 0.84)	−0.66 (−2.80, 1.39)	Cabergoline	4.48 (−1.86, 10.89)	−0.67 (−5.74, 4.39)	3.72 (−3.05, 10.63)	−0.74 (−5.92, 4.45)	1.60 (−4.25, 7.40)	−0.40 (−5.42, 4.63)	0.56 (−4.88, 6.05)	−0.51 (−5.93, 5.10)
	Lazabemide	0.80 (−0.75, 2.33)	1.55 (−0.61, 3.74)	2.22 (−0.53, 4.99)	Lazabemide	−5.20 (−9.77, −0.65)	−0.71 (−7.29, 5.70)	−5.25 (−9.38, −1.09)	−2.92 (−7.70, 1.83)	−4.93 (−9.21, −0.58)	−3.94 (−8.32, 0.47)	−5.00 (−9.53, −0.36)
	Levodopa	−1.62 (−2.74, −0.49)	−0.87 (−2.54, 0.78)	−0.20 (−2.43, 2.03)	−2.43 (−4.33, −0.50)	Levodopa	4.45 (−0.17, 8.91)	−0.04 (−2.55, 2.41)	2.25 (−1.60, 6.14)	0.28 (−2.45, 2.96)	1.30 (−2.09, 4.57)	0.18 (−3.08, 3.53)
UPDRS II	Pergolide	0.17 (−1.90, 2.34)	0.92 (−1.51, 3.37)	1.60 (−1.23, 4.45)	−0.62 (−3.22, 2.02)	1.79 (0.01, 3.63)	Pergolide	−4.50 (−9.56, 0.82)	−2.18 (−8.17, 3.83)	−4.19 (−9.44, 1.24)	−3.18 (−8.73, 2.49)	−4.24 (−9.81, 1.49)
	Pramipexole	−1.60 (−2.33, −0.87)	−0.84 (−2.45, 0.71)	−0.18 (−2.46, 2.14)	−2.40 (−4.09, −0.66)	0.03 (−1.11, 1.17)	−1.77 (−3.92, 0.38)	Pramipexole	2.33 (−1.16, 5.63)	0.32 (−2.06, 2.73)	1.31 (−1.44, 4.03)	0.25 (−2.72, 3.17)
	Rasagiline	−0.42 (−1.69, 0.88)	0.33 (−1.67, 2.28)	1.02 (−1.67, 3.63)	−1.21 (−3.23, 0.79)	1.20 (−0.50, 2.88)	−0.58 (−3.15, 1.88)	1.18 (−0.30, 2.67)	Rasagiline	−2.02 (−5.61, 1.64)	−1.04 (−4.60, 2.78)	−2.10 (−5.83, 1.80)
	Ropinirole	−1.69 (−2.72, −0.67)	−0.93 (−2.74, 0.80)	−0.26 (−2.74, 2.19)	−2.50 (−4.34, −0.64)	−0.08 (−1.47, 1.31)	−1.87 (−4.20, 0.39)	−0.10 (−1.34, 1.10)	−1.27 (−2.95, 0.37)	Ropinirole	0.97 (−1.93, 3.90)	−0.10 (−3.19, 3.09)
	Rotigotine	−1.40 (−2.35, −0.46)	−0.65 (−2.42, 1.12)	0.02 (−2.43, 2.50)	−2.20 (−4.01, −0.40)	0.22 (−1.24, 1.67)	−1.58 (−3.90, 0.72)	0.18 (−0.95, 1.35)	−0.98 (−2.60, 0.61)	0.29 (−1.03, 1.64)	Rotigotine	−1.06 (−4.39, 2.27)
	Selegiline	−1.53 (−2.59, −0.43)	−0.77 (−2.50, 0.97)	−0.10 (−2.50, 2.36)	−2.32 (−4.20, −0.45)	0.11 (−1.37, 1.57)	−1.70 (−4.01, 0.60)	0.06 (−1.22, 1.38)	−1.11 (−2.76, 0.58)	0.17 (−1.28, 1.64)	−0.11 (−1.54, 1.32)	Selegiline
Withdrawals												
	Treatment	Placebo	Bromocriptine	Cabergoline	Lazabemide	Levodopa	Pergolide	Pramipexole	Rasagiline	Ropinirole	Rotigotine	Selegiline
	Placebo	Placebo	1.57 (0.99, 2.45)	1.06 (0.55, 2.11)	0.62 (0.21, 1.93)	0.61 (0.42, 0.92)	1.02 (0.57, 1.88)	1.10 (0.84, 1.46)	0.93 (0.68, 1.29)	0.68 (0.49, 0.97)	0.78 (0.53, 1.13)	1.49 (0.96, 2.39)
	Bromocriptine	−0.36 (−10.69, 10.34)	Bromocriptine	0.68 (0.35, 1.32)	0.40 (0.12, 1.37)	0.39 (0.23, 0.66)	0.66 (0.32, 1.37)	0.71 (0.44, 1.15)	0.59 (0.34, 1.05)	0.44 (0.25, 0.76)	0.49 (0.28, 0.88)	0.95 (0.54, 1.72)
UPDRS total	Cabergoline	—	—	Cabergoline	0.59 (0.16, 2.18)	0.58 (0.31, 1.08)	0.97 (0.41, 2.29)	1.04 (0.52, 2.08)	0.87 (0.42, 1.83)	0.64 (0.31, 1.31)	0.73 (0.34, 1.54)	1.40 (0.65, 3.05)
	Lazabemide	1.78 (−5.25, 8.94)	2.12 (−10.80, 14.80)	—	Lazabemide	0.98 (0.30, 3.19)	1.63 (0.46, 5.73)	1.75 (0.56, 5.54)	1.49 (0.46, 4.78)	1.09 (0.34, 3.46)	1.23 (0.38, 3.94)	2.38 (0.72, 7.84)
	Levodopa	−0.44 (−7.24, 6.71)	−0.11 (−10.99, 11.01)	—	−2.30 (−11.98, 8.12)	Levodopa	1.68 (0.89, 3.15)	1.80 (1.17, 2.75)	1.51 (0.91, 2.51)	1.12 (0.70, 1.75)	1.27 (0.73, 2.14)	2.43 (1.42, 4.23)
	Pergolide	8.19 (−5.83, 22.61)	8.40 (−7.90, 25.00)	—	6.39 (−9.76, 22.48)	8.61 (−4.24, 21.13)	Pergolide	1.08 (0.57, 2.03)	0.91 (0.45, 1.77)	0.67 (0.34, 1.28)	0.76 (0.37, 1.51)	1.46 (0.70, 3.07)
	Pramipexole	−1.00 (−6.66, 5.02)	−0.70 (−11.63, 10.64)	—	−2.81 (−11.78, 6.63)	−0.54 (−6.18, 5.10)	−9.19 (−22.83, 4.50)	Pramipexole	0.84 (0.55, 1.28)	0.62 (0.40, 0.95)	0.70 (0.44, 1.09)	1.34 (0.80, 2.29)
	Rasagiline	−2.89 (−7.66, 1.72)	−2.53 (−14.30, 8.98)	—	−4.67 (−13.32, 3.90)	−2.41 (−11.16, 5.75)	−11.01 (−26.20, 3.79)	−1.89 (−9.74, 5.32)	Rasagiline	0.74 (0.45, 1.18)	0.83 (0.50, 1.36)	1.60 (0.93, 2.82)
	Ropinirole	2.21 (−11.78, 16.68)	2.57 (−14.37, 19.10)	—	0.30 (−15.37, 16.43)	2.62 (−9.84, 14.91)	−5.98 (−23.47, 11.90)	3.19 (−10.28, 16.71)	5.02 (−9.67, 20.27)	Ropinirole	1.13 (0.69, 1.82)	2.17 (1.27, 3.84)
	Rotigotine	—	—	—	—	—	—	—	—	—	Rotigotine	1.93 (1.08, 3.51)
	Selegiline	−6.04 (−11.07, −0.83)	−5.72 (−16.35, 4.83)	—	−7.77 (−16.58, 1.09)	−5.60 (−13.67, 2.23)	−14.16 (−28.85, 0.53)	−5.05 (−12.69, 2.18)	−3.11 (−10.10, 3.83)	−8.24 (−23.07, 6.30)	—	Selegiline

Abbreviation: UPDRS, Unified Parkinson’s Disease Rating Scale.

**Table 4:** Surface under the cumulative ranking curve (SUCRA) results.

	UPDRS II	UPDRS III	UPDRS total	Withdrawals
Placebo	0.214	0.138	0.443	0.445
Bromocriptine	0.440	0.536	0.510	0.093
Cabergoline	0.661	0.613	—	0.403
Lazabemide	0.082	0.097	0.336	**0.762**
Levodopa	**0.749**	**0.761**	0.508	**0.877**
Pergolide	0.205	0.189	0.121	0.427
Pramipexole	**0.738**	**0.777**	**0.565**	0.325
Rasagiline	0.259	0.395	**0.721**	0.531
Ropinirole	**0.773**	0.710	0.369	**0.807**
Rotigotine	0.649	0.536	—	0.704
Selegiline	0.708	0.716	0.918	0.117
