# Supplementary material for: Comparison for Efficacy and Tolerability among Ten Drugs for Treatment of Parkinson’s Disease: A Network Meta-Analysis
Source: Sci Rep. 2017 Apr 4;7:45865. doi: 10.1038/srep45865 (PMC5379205; doi:10.1038/srep45865)
Supplement: Supplementary Information [file srep45865-s1.pdf]

Comparison for Efficacy and Tolerability among Ten Drugs for Treatment of Parkinson's Disease: A Network Meta-Analysis

Running title: Treatment efficacy of Parkinson's disease

Chuanjun Zhuo[1,2,3,4,*], Xiaodong Zhu[5], Ronghuan Jiang[6], Feng Ji[2], Zhonghau Su[7], Rong Xue[5], Yuying Zhou[8]

[1]Department of Psychological Medicine, Wenzhou Seventh People's Hospital, Wenzhou, 325005, Zhejiang, China

[2]Institute of Mental Health, Jining Medical University, Jining, 272067, Shandong, China

[3]Department of Psychological Medicine, Tianjin Mental Health Center, Tianjin Anding Hospital, Tianjin, 300222, China

[4]Department of Psychological Medicine, Tianjin Anning Hospital, Tianjin, 300222, China

[5]Department of Neurology, Tianjin Medical University General Hospital, Tianjin, 300075, China

[6]Department of Psychological Medicine, Chinese PLA (People's Liberation Army) General Hospital, Beijing, 100853, China

[7]Department of Psychological Medicine, The Second Affiliated Hospital of Jining Medical College, Jining, 272100, Shandong, China

[8]Department of Neurology, Tianjin Huanhu Hospital, Tianjin Brain Center, Tianjin, 300350, China

***Correspondence to:** Chuanjun Zhuo, Department of Psychological Medicine, Wenzhou Seventh People's Hospital, No.522 Xinshan East Road, Wenzhou, Zhejiang, 325005, China, Email: zhuxiuhui1971@163.com; Tel./Fax: +86- 577-88414131

Supplementary Figure Legends

Figure S1. Flow diagram of study selection.

Figure S2. Forest plot for mixed treatment comparison of UPDRS II with mean difference (MD) and 95% confidence interval (CI).

Figure S3. Forest plot for mixed treatment comparison of UPDRS III with mean difference (MD) and 95% confidence interval (CI).

Figure S4. Forest plot for mixed treatment comparison of UPDRS total with mean difference (MD) and 95% confidence interval (CI).

Figure S5. Forest plot for mixed treatment comparison of withdrawals with mean difference (MD) and 95% confidence interval (CI).

Figure S6. SUCRA ranking table with respect to UPDRSII, UPDRSIII, UPDRS total and withdrawals The ranking is based on surface under the cumulative ranking curves (SUCRA) values, the higher value means the better ranking.

Figure S7. Results of the node splitting method. P-value means the consistency of direct and indirect evidence, p>0.05 means there is no significant inconsistency.

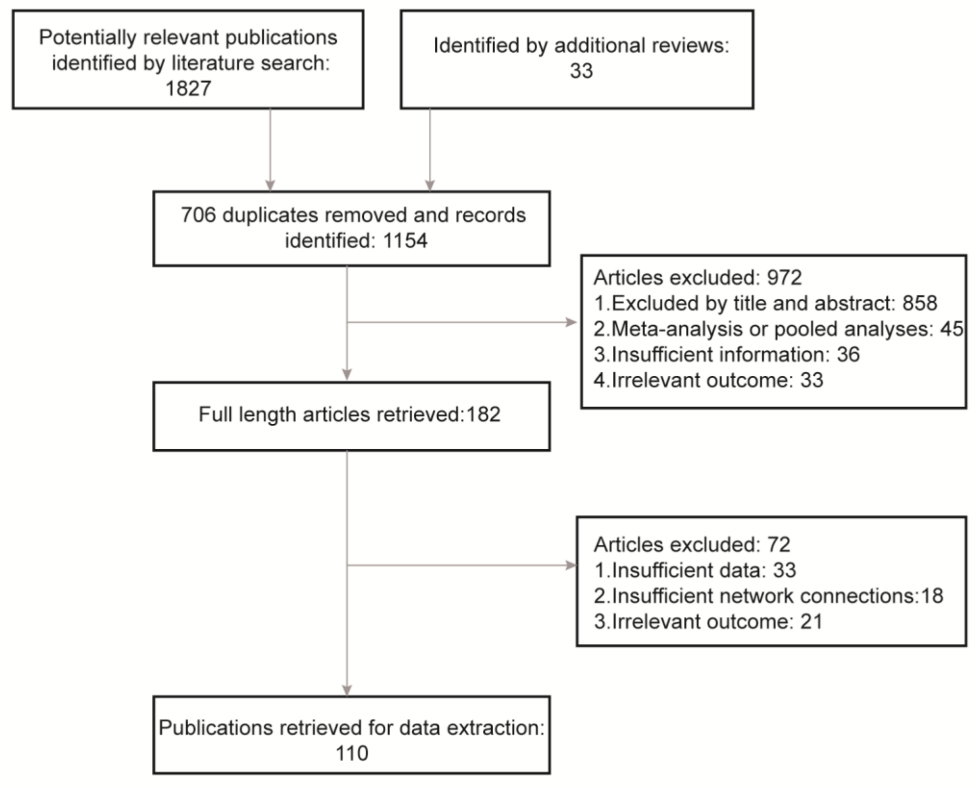

Figure S1. Flow diagram of study selection.

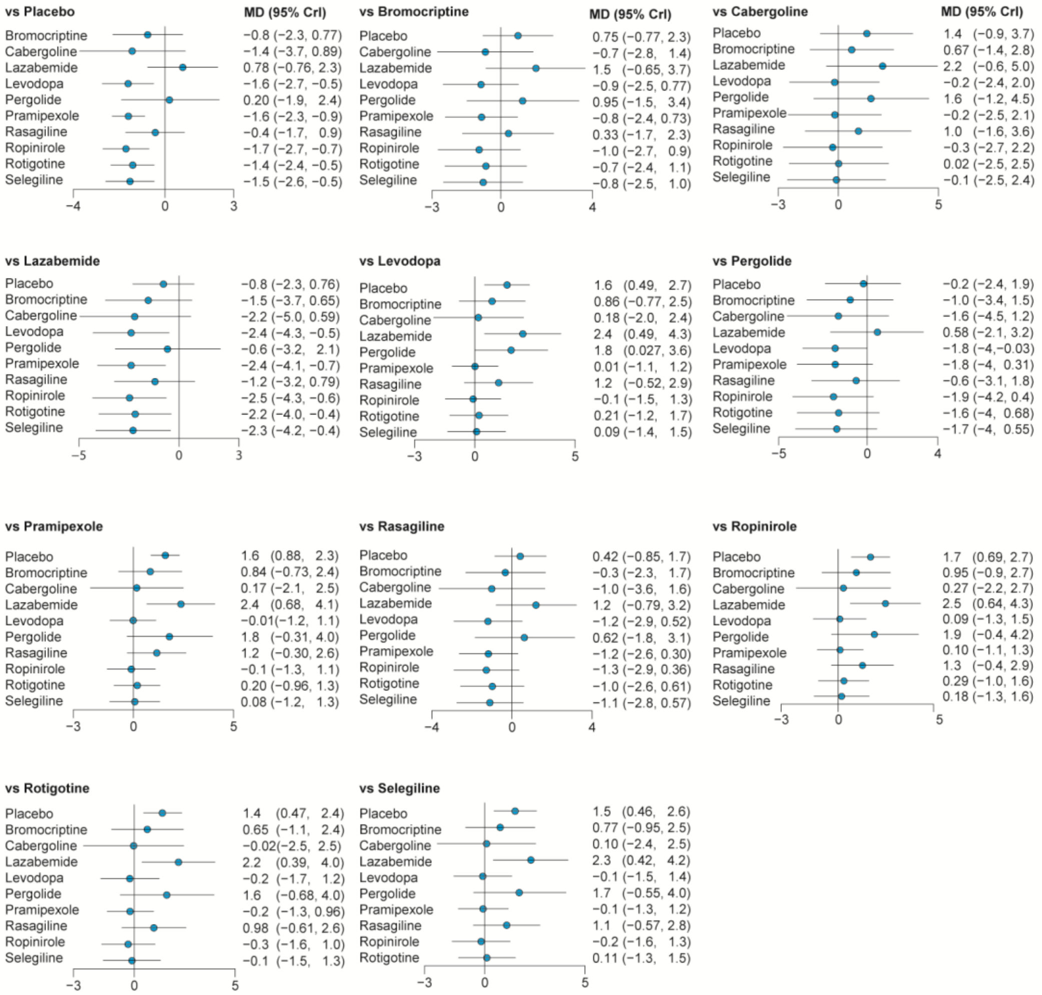

Figure S2. Forest plot for mixed treatment comparison of UPDRS II with mean difference (MD) and 95% confidence interval (CI).

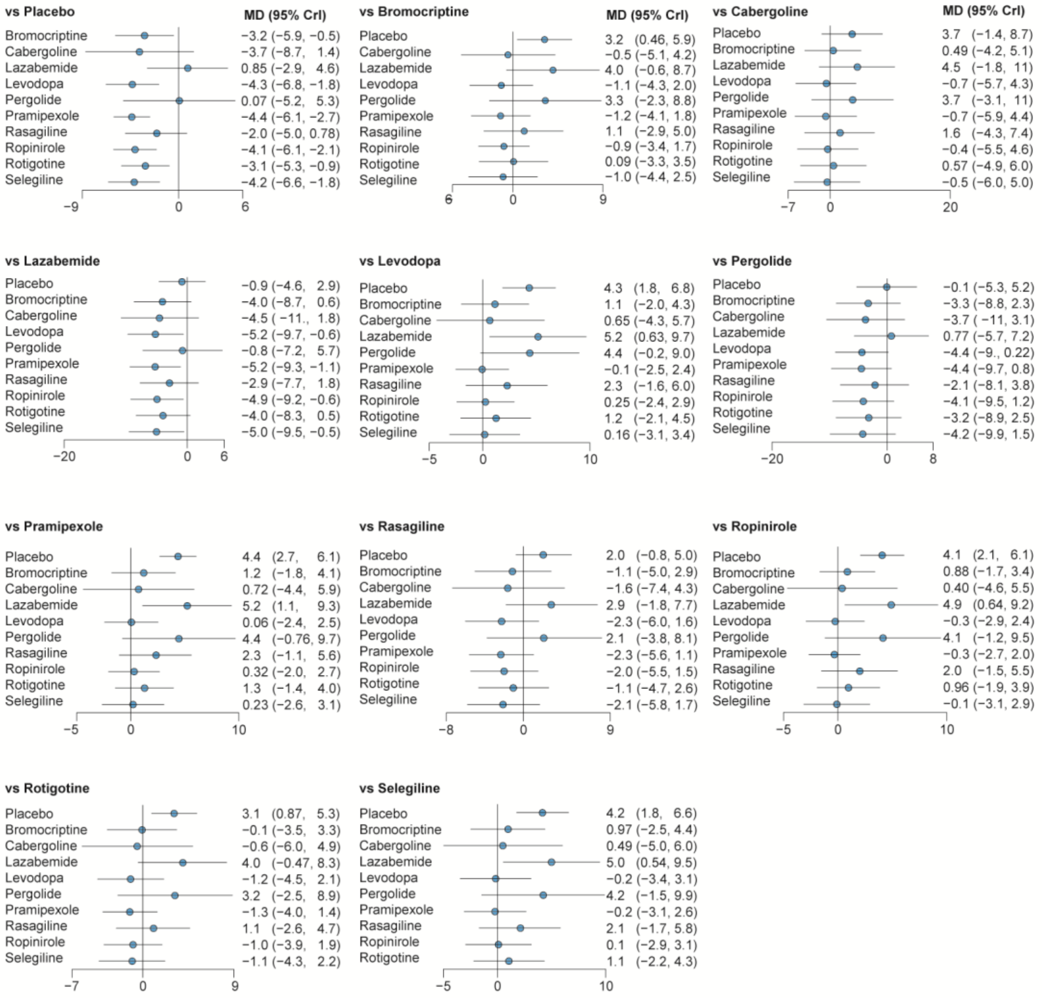

Figure S3. Forest plot for mixed treatment comparison of UPDRS III with mean difference (MD) and 95% confidence interval (CI).

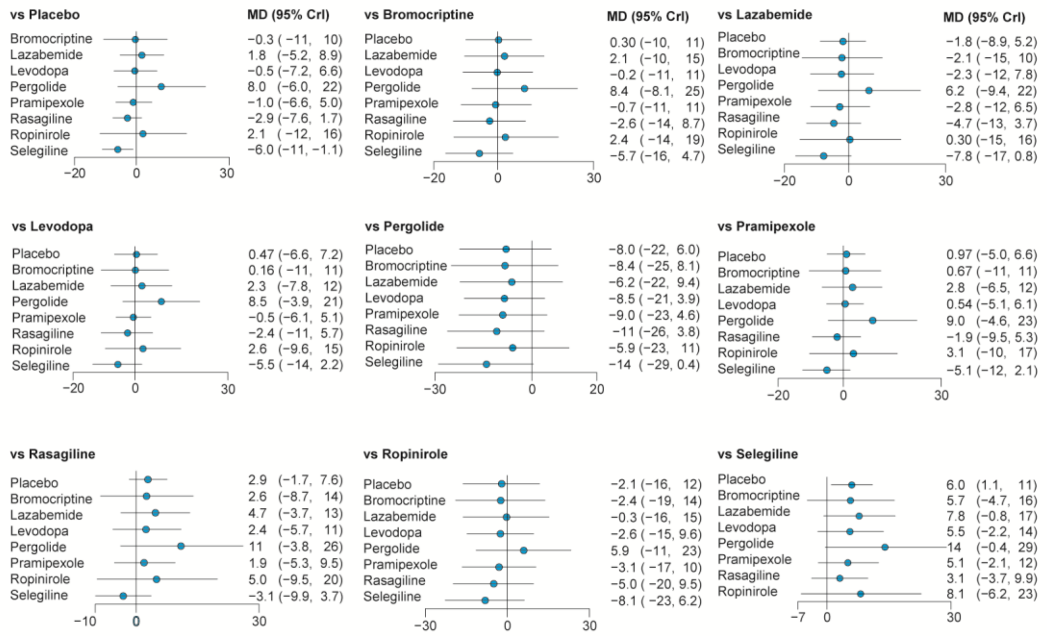

Figure S4. Forest plot for mixed treatment comparison of UPDRS total with mean difference (MD) and 95% confidence interval (CI).

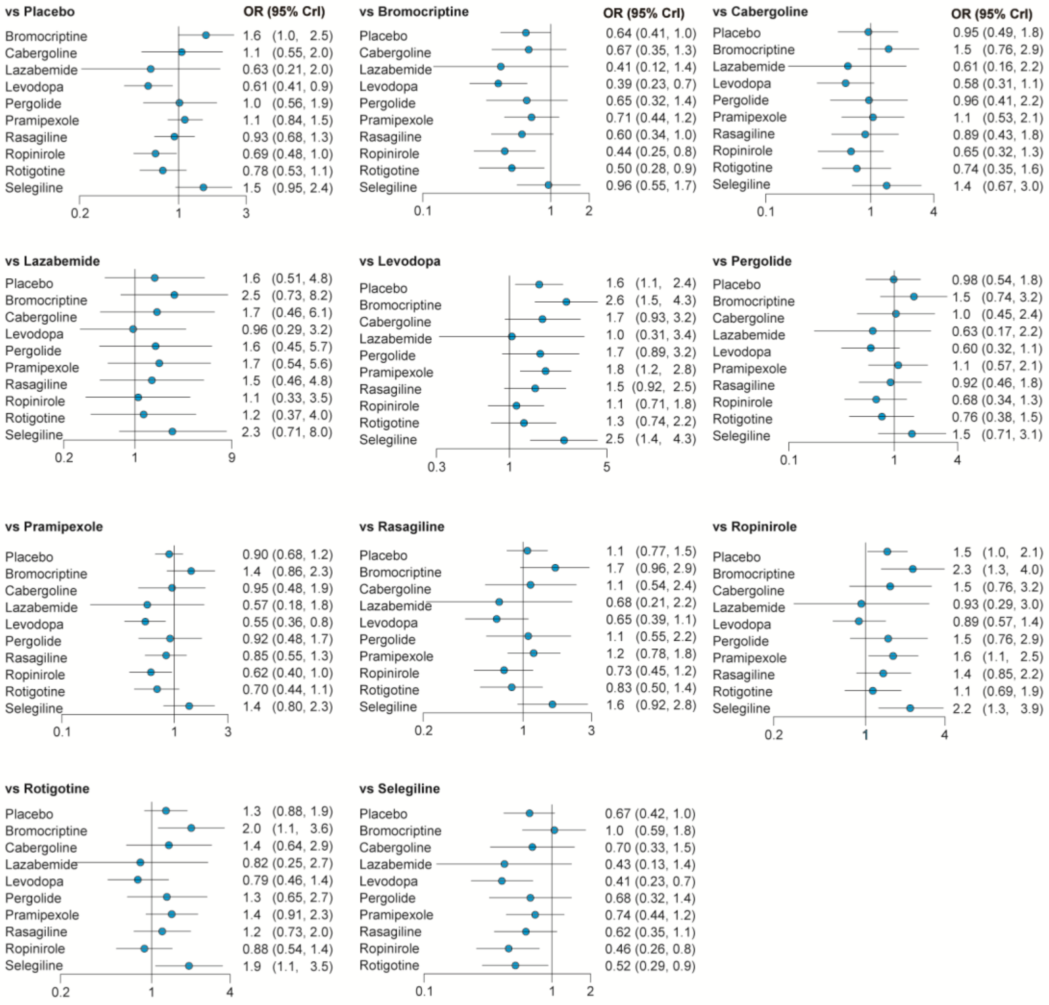

Figure S5. Forest plot for mixed treatment comparison of withdrawals with mean difference (MD) and 95% confidence interval (CI).

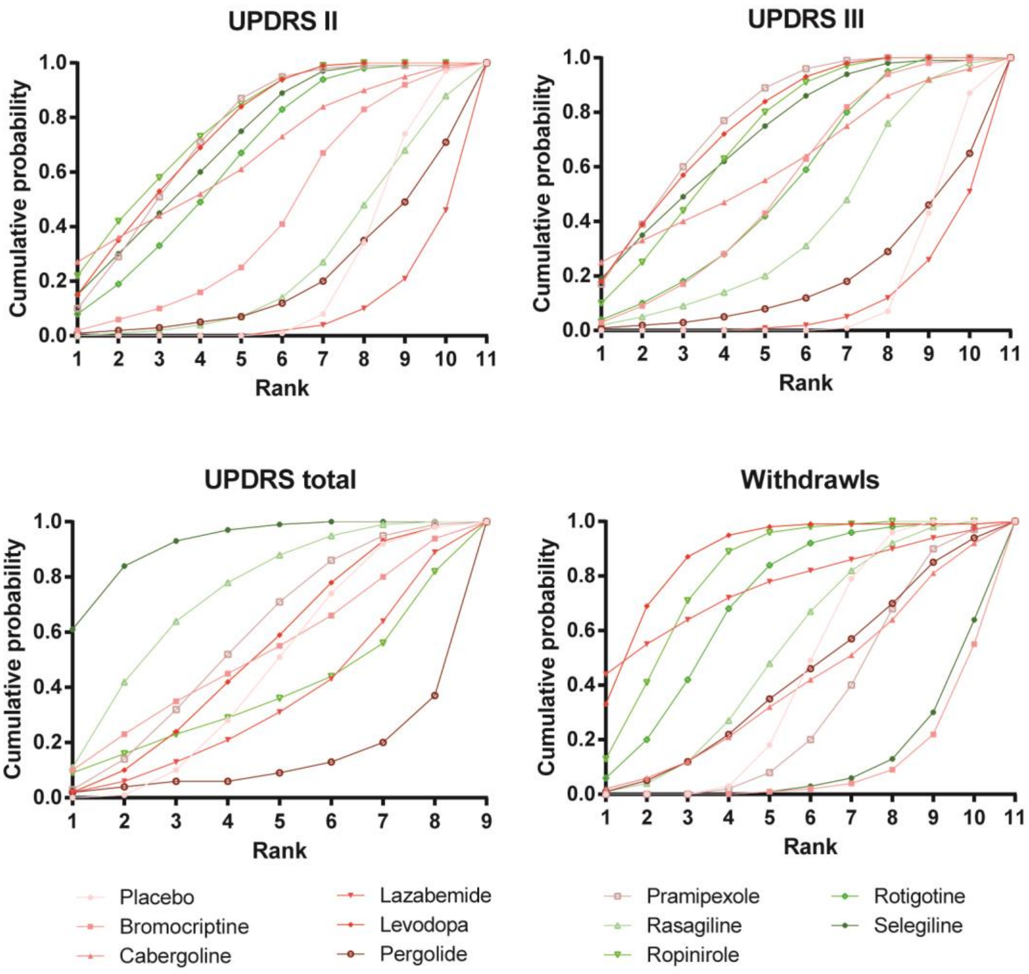

Figure S6. SUCRA ranking table with respect to UPDRSII, UPDRSIII, UPDRS total and withdrawals The ranking is based on surface under the cumulative ranking curves (SUCRA) values, the higher value means the better ranking.

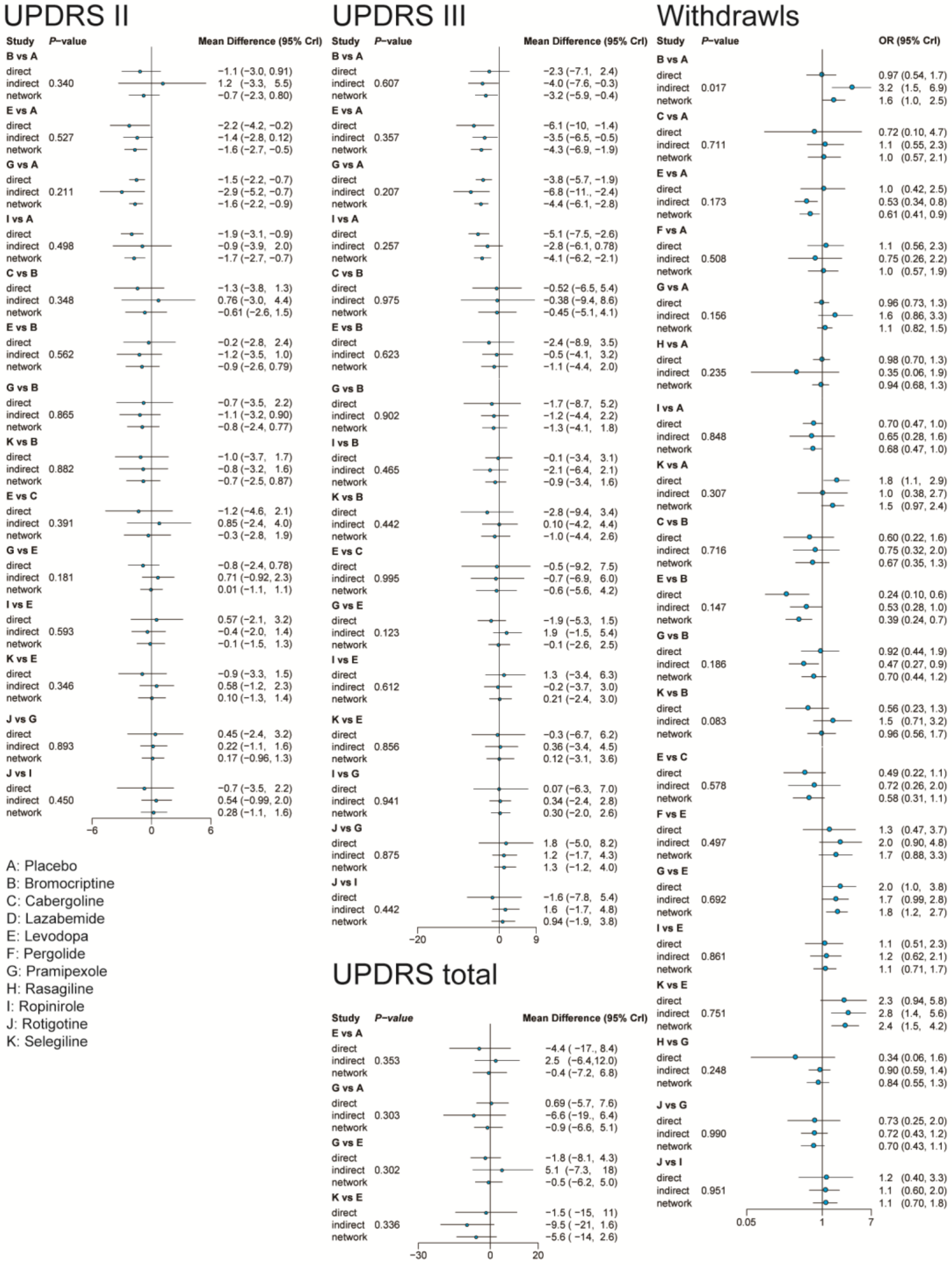

Figure S7. Results of the node splitting method. P-value means the consistency of direct and indirect evidence, p>0.05 means there is no significant inconsistency.
